# The Paris Declaration in practice: challenges of health sector aid coordination at the district level in Zambia

**DOI:** 10.1186/1478-4505-7-14

**Published:** 2009-06-08

**Authors:** Jesper Sundewall, Birger C Forsberg, Kristina Jönsson, Collins Chansa, Göran Tomson

**Affiliations:** 1 Division of Global Health (IHCAR), Karolinska Institutet, Sweden; 2 Ministry of Health, Lusaka, Zambia; 3 Centre for East and Southeast Asian Studies, Lund University, Scheelevägen 15D 223 63, Lund, Sweden; 4 Division of Global Health (IHCAR) and Medical Management Centre, Karolinska Institutet, Sweden

## Abstract

**Background:**

The increasing resources available for and number of partners providing health sector aid have stimulated innovations, notably, the Paris Declaration on Aid Effectiveness, which aim to improve aid coordination. In this, one of the first studies to analyse implementation of aid coordination below national level, the aim was to investigate the effect of the Paris Declaration on coordination of health sector aid at the district level in Zambia.

**Methods:**

The study was carried out in three districts of Zambia. Data were collected via interviews with health centre staff, district managers and officials from the Ministry of Health, and from district action plans, financial reports and accounts, and health centre ledger cards. Four indicators of coordination related to external-partner activity, common arrangements used by external partners and predictability of funding were analysed and assessed in relation to the 2010 targets set by the Paris Declaration.

**Findings:**

While the activity of external partners at the district level has increased, funding and activities provided by these partners are often not included in local plans. HIV/AIDS support show better integration in planning and implementation at the district level than other support. Regarding common arrangements used for fund disbursement, the share of resources provided as programme-based support is not increasing. The predictability of funds coming from outside the government financing mechanism is low.

**Conclusion:**

Greater efforts to integrate partners in district level planning and implementation are needed. External partners must improve the predictability of their support and be more proactive in informing the districts about their intended contributions. With the deadline for achieving the targets set by the Paris Declaration fast approaching, it is time for the signatories to accelerate its implementation.

## Introduction

With increases in the number of donors and resources available, as well as the broadening diversity of aid projects in the past 20 years,[1,2] growing attention has been paid to optimizing the effectiveness of international aid, particularly in the health sector, and has highlighted the need for improvements in the coordination of donor efforts [3,4]. The Paris Declaration on Aid Effectiveness, endorsed in 2005, is an international agreement with 130 signatories (including more than 100 countries and organizations), which calls for increased harmonization, alignment and management of aid and sets out indicators by which results can be monitored [5]. Targets for most of these indicators were set to be achieved by 2010.

The importance of coordination and predictability of donor aid in the health sector has also been emphasised by the recently launched International Health Partnership [6] – a coalition of international health agencies, governments and donors committed to improving health and development outcomes in developing countries and achieving the Millennium Development Goals related to health. Coordination has also been one of the main arguments for aid modalities such as sector-wide approaches (SWAp) and general budget support [7-10].

Between 1994 and 2007, donor coordination was the subject of 21 published peer-reviewed articles on health policy [11]. Few of these articles, however, studied how donor coordination is implemented at the national or the district level and the benefits of coordination remain unclear [12,13]. A follow-up of the Paris Declaration indicators at the national level, initiated by the Organisation for Economic Co-operation and Development (OECD), suggested that, at the current pace of progress, the 2010 targets will not be met. In Zambia the OECD observed progress at national level as aid is becoming more predictable and more often reported on national budgets. On the other hand, the OECD survey noted lack of progress in other indicators; the number of parallel implementation units has increased in Zambia and the share of support which is programme based has not grown [14]. However, implementation at lower levels remains largely unexplored. Although policy theory suggests that implementation of public policy mainly takes place at lower levels (e.g. districts) [15,16], none of the abovementioned articles give a detailed account of implementation at district level.

In the present study, the aim was to investigate the effect of the Paris Declaration on coordination of health sector aid at the district level in Zambia. Defined in this study as "joint donor and government planning and implementation of activities and funds", coordination in three selected districts was analysed from a policy-implementation perspective using four indicators. The indicators were then assessed in relation to the targets set out in the Paris Declaration.

In Zambia, which has a long experience of aid coordination, the goal is to coordinate all resources and activities under the SWAP framework. Aid to the health sector is provided through a mix of different mechanisms; as stand alone-projects, through pooled funding mechanisms and as general budget support. Regardless of how resources are channelled, however, the activities or components supported should be in line with the priorities stated in the National Health Strategic Plan. Over the last ten years, the amount of resources available to health has increased substantially and in 2007 total donor contributions amounted to approximately USD 350 million (Ministry of Health: External finances in the health sector, unpublished).

**Figure 1:**
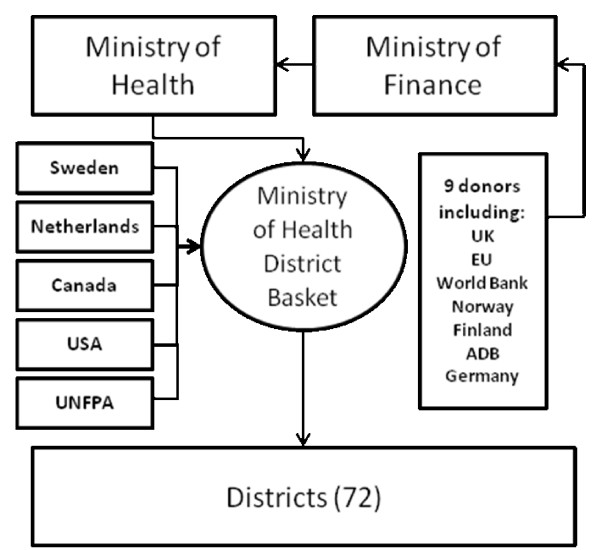
**Overview of the district basket funding mechanism in the Zambian health sector**.

Coordination of aid in the health sector in Zambia has been on the agenda since the early 1990s when the health sector reforms were introduced. With the reforms came the introduction of a joint donor/government mechanism for financing of health services at the district level, the so-called "district basket". The purpose of the district basket and the decentralised approach was to increase the amount of resources channelled to the district level and to improve district autonomy with regard to planning and setting of priorities [17]. Later the basket was expanded to also finance other parts of the health system, although support to district health services still constitutes the main part of the expanded basket. In 2007 direct contributions to the basket were provided from a handful of donors (Sweden, the Netherlands, Canada, the United States and UNFPA) and the Government of Zambia. The Government contribution to health increased in the last couple despite some of the previous contributors to the basket (the European Union and The United Kingdom) moving over to budget support and thus channelling their assistance via the Ministry of Finance to the Ministry of Health. Figure 1 provides an overview of how resources are channelled through the district basket showing the main contributors.

The main responsibility for the delivery of primary health-care in Zambia lies with the districts. There are processes in place for "bottom-up" planning; from the district to the national level. Following specifically developed planning handbooks, districts, health centres and district hospitals develop annual action plans with guidance from the district health management teams and these feed into the consolidated action plan at the district level. The planning handbooks provide detailed instructions as to how to conduct the planning but also on how the district grants can be used specifying minimum and maximum spending for each level of the district; community, health centre, district hospital and district office [18]. At the launch of the planning cycle, districts are given an indicative planning figure which gives guidance to the districts as to how much funds they will receive for the coming year from the Government and the district basket. The allocations to the districts follow a refined formula taking into account socio-economic factors, material deprivation and disease burden. Once planning is completed, the provincial health office assesses the plan through a peer review process and changes are suggested and negotiated.

Coordination of health sector aid at the district level is the task of the district health teams, who should "lead and coordinate the work of local non-governmental organizations and other stakeholders in the district" [19]. Formal structures for coordination of partners at district level are, however, limited. External partners should ideally be invited to the planning activities by the districts and their proposed activities should be incorporated in the district action plan. At the national level, donor resources are coordinated under the Zambian health SWAp [20]. The Ministry of Health and donors meet regularly in donor-group meetings, SWAp meetings and technical working groups. The Zambian health SWAp has strong support among both the government and donors. Achievements have been recognized by both partners as formal structures for coordination, harmonization and alignment have been established and are increasingly well functioning [21]. However, the ability of the SWAp to contribute to more efficient resource allocation at the national level is yet to be proven [12]. Results in terms of access to services and health outcomes are mixed. Drug availability and immunization coverage has improved while there has been limited decline in disease burden indicators [21].

## Methods

Three districts of Zambia were selected: Kabwe, Kafue and Mumbwa, one urban, one peri-urban and one rural district. While the number of management staff, population and number of health facilities was similar in each, the districts differed in terms of population, burden of disease, resources available and management capacity. In order to get a sample of districts illustrating these differences, guidance for the selection was provided by the Ministry of Health Directorate of Planning and Development after an extensive review of data from the health-management information system and the medium-term expenditure framework. Ethical approval for the study was obtained from the University of Zambia research ethics committee.

The four indicators studied – which covered the themes of activity of external partners, common arrangements or procedures and predictability – were selected from the Paris Declaration and adapted to fit the district context in Zambia. A summary of the indicators used and main sources of data accessed is given in Table 1. District funding in Zambia consists of three main components: grants from donors and the Ministry of Health, user fees in medical facilities and so-called *"other" income*. The grants from the donors and Ministry of Health, hereinafter referred to as *district grants*, are provided jointly through the basket funding mechanism. In this study, user fees are grouped together with "other" income at district level. User fees were abolished in all rural areas of Zambia in 2006 and both Mumbwa and Kafue are classified as rural districts.

**Table 1:** Areas of coordination, indicators and data sources used to investigate donor coordination at the district level in Zambia

**Coordination areas**	**Indicators**	**Main data source**
Activity of external partners	Number of partners and their involvement in planning	District actions plans and interviews
Use of common arrangements or procedures	Share of resources to districts provided as programme-based support (resources coordinated under the Zambian health SWAp)	District action plans, financial reports, bank statements and interviews
Predictability	Actual financial resources at district level as a percentage of total resources budgeted for Regularity of district disbursements to health centres	District action plans, financial reports, bank statements and interviews Health centre ledger cards, receipts and interviews

SWAp, sector-wide approach

Indicators adapted from the Paris Declaration, 2005

Resources provided as *programme-based support *was in this study defined as resources channelled through the district grants and all remaining resources were considered as "other" income. This definition of programme-based approach differs from the OECD definition which relies on a more qualitative assessment. According to OECD, programme based support is provided under a recipient led, comprehensive programme framework having formal processes for donor coordination [14].

Data collection was conducted during November 2007 – February 2008. A total of 22 semi-structured interviews were conducted with one or two staff members in each health centre, three managers in each district and five Ministry of Health officials. The interviews followed an interview guide that was specific to each level of inquiry. Respondents were encouraged to elaborate freely on their responses from their personal experience. All interviews were conducted by the first author of this article at the respondent's place of work and lasted an average of 45 minutes. The appropriateness and practical application of the interview guides was tested in four pilot interviews with district representatives and staff from the Ministry of Health.

After review of the action plans for all districts, a list of all external partners was compiled (excluding government departments and other line ministries mentioned in the action plans). District financial receipts from the Ministry of Health were analysed through a review of bank statements, financial reports and action plans. The district accountant assisted in interpretation of items and codes in the documents. Local-level data were collected by reviewing cash books and ledger cards for two health centres in each district with the assistance of local accountants or the person "in charge". Health centre cash-books were matched with payment receipts. Relying on multiple data sources was useful as data at the district level was sometimes limited. It allowed for cross-checking of findings in order to increase validity and reliability. Information from the review of financial data was also followed-up in interviews for further clarifications. All districts and health centres provided unrestricted access to their financial archives. Quantitative data was compiled and analysed using Microsoft Excel. The interviews were used to complement and further explain the quantitative data and followed an "embedded" analysis where specific subunits of each case were further explored [22].

## Results

### Activity of external partners

**Figure 2:**
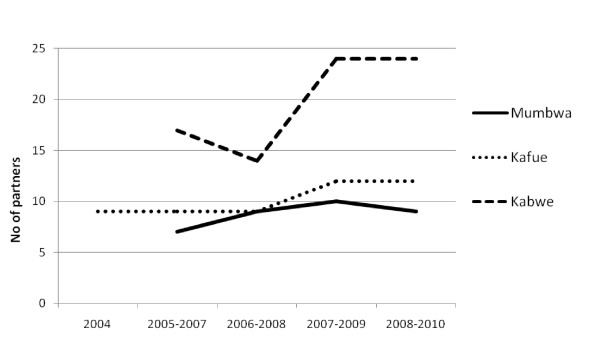
**Number of donors, non-governmental organizations and civil-society organizations listed as partners in three districts in Zambia, from district health-sector action plans for 2004–2008**.

The findings of this study showed that between 7 and 24 external partners were working in the health sector in each of the three selected districts in Zambia during the last five years (Figure 2). In all districts, the number of partners has grown over the last four years, in one district by as much as seven partners. This increase is mostly due to new partners working in the area of care and treatment for people with HIV/AIDS, according to information obtained from the interviews.

Partners at district level are rarely the same at national level as few bilateral and multilateral donors have direct presence at district level, the Japanese International Cooperation Agency – JICA – being one of few exceptions. Instead partners at district level are mainly NGOs and Civil Society Organizations working in specific areas, for example HIV/AIDS or infrastructure development. Funding for district level partners, however, often come from bilateral or multilateral agencies working at national level. For example NGOs working in HIV/AIDS are often funded by the US Government through the PEPFAR (President's Emergency Plan for Aids Relief) Program or the United States Agency for International Development, USAID.

According to the results of interviews with district and health centre staff, partner involvement in the district planning process was limited in all three districts. In none of the three districts, partners were involved in the annual planning process. District respondents expressed the view that it was difficult to include partners in planning as their activities were often predetermined and districts had little power over the priority-setting of funds and activities of the partners. For specific activities, however, there were several examples of partner involvement.

*"For child health week we had a special meeting where we brought all stakeholders to the table and different partners pledged different support. Some pledge to provide fuel, others food and so on. We do similar things for the international AIDS day." *(District Director of Health)

According to respondents one difficulty with this approach is that partners often propose support for specific activities that districts could only reject or approve, and partners were often unable to predict the amount of funds or activities that they would provide. This is one explanation for the fact that these resources are not reflected in the district budgets. According to interviews, HIV/AIDS partners seemed to be more integrated with respect to planning and provision, although their involvement was still limited. One district argued that an example of better integration in service provision is the fact that much of the curative services in HIV/AIDS are provided in government health facilities, but with specific extra support from partners. Another district reported that there was regular planning sessions with one partner in HIV/AIDS.

*"Our partner in HIV provides material, training etc. Normally they come every two weeks and we have a very good dialogue with them." *(District Director of Health)

According to a Ministry of Health respondent it is a problem that partner contributions are not captured in the district action plans. Furthermore, the fact that "other" income is not disaggregated in the action plans means that the Ministry of Health does not know what these funds cover as they can be anything from housing allowances provided by the Ministry of Health to funds for a specific activity from, for example, the Global Fund. A Ministry of Health respondent argued that a strict interpretation would mean that partners whose activities are not included in the districts action plan should not work in the district. At the same time, the Ministry is well aware of this being a common occurrence.

### Common arrangements or procedures

Support to districts which is provided as programme-based support includes resources under common, SWAp-type arrangements which are aligned with country-led strategic plans. It is assumed that the higher the share of programme-based support, the more aid is coordinated in line with the National Health Strategic Plan.

**Figure 3:**
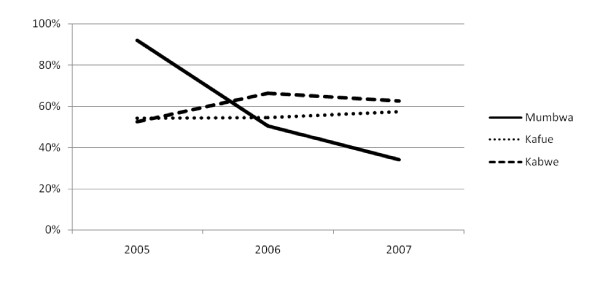
**Share of financial resources in district action plans provided as programme-based support for three districts of Zambia, 2005–2007**.

In Mumbwa, the share of resources provided as programme-based support has declined for the last three years and is now less than 40% (Figure 3). In the other two districts, the share has remained fairly constant at between 54% and 66%. The decline in Mumbwa is attributable mainly to a large increase of "other" income, including considerable contributions for the construction of a new district hospital which was disbursed in 2006 and 2007. "Other" income grew in the other two districts as well, but it was matched by a concurrent increase in resources provided as programme-based support.

### Predictability

Predictability of foreign assistance generally means that donors should provide more long-term indicative figures of how much aid they will provide but also to disburse aid commitments in a timely manner [14]. Predictability of resources is recognised as an important factor for countries to be able to manage public financing and undertake realistic planning.

**Figure 4:**
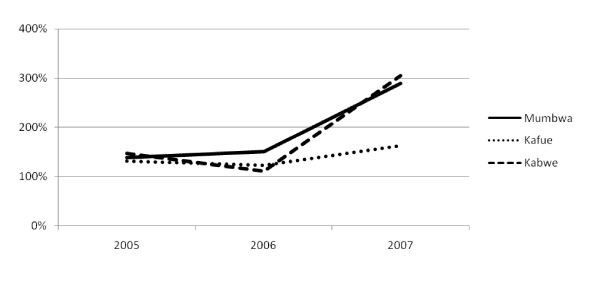
**Actual financial resources as a percentage of planned resources at district level in three districts in Zambia, 2005–2007**.

As seen in figure 4, data from action plans revealed that, in all three districts, actual financial disbursements were constantly above 100%, i.e. more resources arrived than were planned for. In two districts, disbursements in 2007 had reached levels of about 300% of the planned resources.

Further disaggregation showed that funds from the district grants were disbursed more predictably than "other" income. Average disbursements from the district grants to the three districts were 98%. Kafue had the lowest average disbursement at 85% and Kabwe the highest at 110%. Respondents stated that the arrival of district grants was mostly timely. This was confirmed by bank statements. Interviewees claimed that the predictability of district grants was often good, although extraordinary events such as the appreciation of the *kwacha *(the currency of Zambia) in late 2005 rapidly reduced the true value of the grant.

*" [District] grants are regular but sometimes come too late in the month. If they came in the beginning of the month which they were supposed to cover it would be better." *(District Director of Health)

Average disbursements of "other" income, however, reached more than 1500% of the planned level. Respondents explained the numbers by the fact that only funds that can be confirmed by the district are included in the action plan, and the predictability of most income labelled as "other" is not known when action plans are compiled.

Predictability at the health centre level was assessed by reviewing the regularity of cash disbursements to health centres (the so-called *imprest*, cash payments to districts to meet operational costs). The sum given as imprest is decided on a monthly basis by the district office, based on the grant received from national level. Health centre staff stated that the imprest was not always disbursed as planned. This was confirmed by health centre ledger cards, which showed that imprests were sometimes paid irregularly and also showed large variations in the amounts disbursed (Table 2). Interviews with health centre staff highlighted the fact that health centres are not able to influence the amount received as monthly imprests.

**Table 2:** Imprests received by health centres in three districts of Zambia, 2006 and 2007

**Month**	**District**											
	**Kafue**				**Mumbwa**				**Kabwe**			
	
	Health centre 1		Health centre 2		Health centre 1		Health centre 2		Health centre 1		Health centre 2	
	
	2006	2007	2006	2007	2006	2007	2006	2007	2006	2007	2006	2007

January	1000	1086	1080	607	--	--	--	300	1709	420	--	660
February	1000	1129	--	978	1000	200	1000	300	--	420	--	600
March	--	1172	1080	--	--	--	--	386	--	420	--	1000
April	--	1172	--	1226	--	400	--	--	--	600	--	2700
May	--	1172	700	--	600	590	600	400	--	--	1000	1224
June	255	1172	--	1458	600	600	600	400	400	--	--	2125
July	--	1172	580	--	--	--	--	--	--	--	--	--
August	455	1172	--	869	750	400	400	600	423	464	660	2125
September	348	1472	450	--	--	--	--	300	--	1200	--	2100
October	348	1472	781	1172	480	650	480	280	420	--	2320	1840
November	3206	1472	1281	781	300	--	300	--	420	1000	--	--
December	1892	1472	781	391	700	400	--	350	--	980	660	--
Total	8504	15135	6733	7482	4430	3240	3380	3316	3372	5504	4640	14374

Imprest, cash payments to health centres

*The funding fluctuates depending on the idea of [district] management. Sometimes they purchase things on our behalf and sometimes they give us money*. ("In-Charge" rural health centre)

During 2006, imprests were paid in 4–8 months of the year in the six health centres visited during the study, while in 2007 imprests were paid more frequently, in 7–12 months, and the total amount disbursed had also increased in four out of six centres. Only one health centre received an imprest every month.

## Discussion

The results of our study show that aid coordination at the district level in Zambia is weak. Although there is a consultative process for planning, partner involvement in this process is limited. A high proportion of partner funds and activities are not included in local plans and budgets. Nevertheless, government policies and strategies, as well as the targets of the Paris Declaration, call for all funding to be aligned with national priorities [5,23,24].

To our knowledge, this is the first published study to assess the implementation of aid coordination in the health sector below the national level. We chose to approach the subject from the perspective of the Zambian government. A partner perspective might give a different view of the extent to which resources are coordinated, not least because operational definitions of key concepts may vary from actor to actor. For this study, however, the government's perspective was deemed the most relevant as they are responsible for the coordination of aid in the health sector. Although external partners are involved in the coordination process, it is the government which is ultimately held accountable for failure or success in the implementation of the annual health sector action plans.

The number of partners at the district level in Zambia has increased. This is similar to the global development where an increase in bilateral donors and non-governmental organisations has been observed [1]. New "emerging" donors have also become active, especially in the areas of HIV/AIDS, tuberculosis and malaria [25,26]. The increase in partners at the district level is, however, in conflict with the national level aim in Zambia, which is to leverage the comparative advantage of each donor by reducing the number of donors involved in each sector. The reduction is encouraged by promoting delegated partnership, i.e. one donor channeling its support through another donor's administrative mechanism. It is the main goal of the Joint Country Assistance Strategy for Zambia, a framework agreed between donors and government in Zambia to detail how all aid, irrespective of sector, should become more country-led, aligned and harmonized – all in the spirit of the Paris Declaration [24]. If a reduction of partners at the national level is replaced by an increase of partners at district level it is unlikely that external assistance will become more recipient-led, aligned and harmonized at lower levels of the health system. Such a development seems contradictory to the intentions of both the Paris Declaration and the Government of Zambia.

Unexpectedly, interviews showed that funds and activities organized by HIV/AIDS partners appear to be more integrated in district planning than are the activities of district partners overall, which are poorly integrated in this process. Explanation for this may lie in the fact that aid programmes financed through global health initiatives, such as those for HIV/AIDS, more frequently lack coordination at the national level [25] and HIV/AIDS control programmes have often been criticised for being too disease-specific and not aligned with national planning and implementation; this criticism itself may have encouraged such programmes to adapt and change. Furthermore, though not explored in this study, it could well be that partners working in HIV/AIDS are better financed than other organizations. The large increase in donor funding for health in Zambia in recent years have predominantly been for HIV/AIDS related work. Therefore, organizations implementing HIV/AIDS activities might have more resources and capacity to devote time for coordinating their activities with the district office. It could also be argued that there is more to gain for districts in coordinating partners with large resources than those with few resources, and this could explain their better integration in district planning and service provision.

Our findings do not suggest that the donors are sufficiently relying on common arrangements. The target of the Paris Declaration is that 66% of all aid should be provided as programme-based support by 2010. Programme-based support in all three districts in this study was below this target and there was no upward trend – in fact, in the district of Mumbwa there had been a sharp decline in the last three years. However, on a positive note, when compared with baseline data from the OECD, the share of programme-based support in the three districts selected was higher than the national average; in 2006, for example, only 47% of all aid to Zambia was programme-based [27]. Our results, however, are likely to be an overestimation of the share of resources provided as programme-based support as we have defined such support as resources provided as basket grants. Evidently, non-programme based resources are also coordinated within the Zambian health sector, but interviews indicate that those are rarely planned for or coordinated at the district level. At best they are reflected in the accounts at the end of each year. If those resources were fully included in the district budget, the share of programme-based support would be reduced.

The predictability of the regular government district grants was high as such funds were disbursed regularly and disbursement levels were close to budget targets during the most recent years. Predictability of "other" income was, however, more off budget. In all three districts, the level of "other" income by far exceeded the budgeted amounts. The Paris Declaration target to increase aid predictability is that 87% of aid should be disbursed as scheduled by 2010 [5]. In a 34-country comparison undertaken by the OECD in 2006 in order to monitor the effect of the Paris Declaration, the average ratio of aid disbursed in a timely fashion was 41%, and this value was 50% in Zambia [27]. Previous studies have suggested that effective planning and implementation requires predictability of funds and activities [28,29]. Long-term donor commitment with increased predictability of resources is also an explicit aim of both the Paris Declaration and the sector-wide approach model [5,30]. There is an apparent risk of activities being rescheduled, cancelled or not even planned when the availability of resources at the district level is not well known. Furthermore, according to interviews, Ministry of Health regulations give districts little freedom to prioritise grants, and even less power over prioritising "other" income as these are normally provided for an earmarked purpose. This suggests that power over planning and priority setting at the district level is weak. Similar observations have been made in the Ugandan health sector, where power over planning became increasingly centralised when the SWAp was introduced [31].

We found that the imprest payments (cash payments) from districts to health centres were not disbursed on a monthly basis as scheduled. Owing to irregular payments, implementation of activities at the district level seemed to be determined more by the time at which funds became available, than by when they were most needed. Other studies have also identified constraints for local level implementation, for example shortages in human resources, which exist at all levels of the Zambian health system [32,33]. Furthermore, conditions imposed by donors and the government limit the decentralization of planning and implementation of services [34].

The district of Mumbwa had the highest share of funds not budgeted for and the lowest share of resources provided as programme-based support. At the same time, Mumbwa was the district with the fewest external partners. The main explanation found was that the action plan for Mumbwa included hardly any "other" income, although receipts in Mumbwa were substantial for all three years studied. Despite the fact that the number of external partners was by far the highest in Kabwe, Kabwe had a higher share of support that was programme-based than Mumbwa and better predictability of resources. One explanation could be that partners' contribution was relatively small. Another possibility, which is perhaps more likely, is that the partners' contributions were not recorded or quantified, neither in plans nor in follow-up, indicating that the share of programme-based support in Kabwe might be lower than results show.

Coordination efforts have often focused on the national level and participation at lower levels has been limited [34]. The limited involvement of partners in coordination at the district level can lead to planning and implementation becoming more ad hoc and this is supported by our results. At the same time, it has been proposed that planning of health services and resource use in a decentralised health system should be from the bottom up [35]. The decentralised approach in the Zambian health sector was also designed to strengthen planning and implementation at the district level [17]. Districts in Zambia are supposed to set their own priorities, but our results show that they have limited ability to control how to fund these priorities, suggesting that there is limited local level autonomy.

The overall goals of coordination and decentralization are fully compatible but potential areas of conflict exist. The larger the number of decision makers involved the more complicated and challenging coordination becomes. A balance must be struck between allowing local autonomy and imposing requirements from the national level [36]. It seems that for local priority setting to evolve in the districts studied, more local control over funds is required.

Zambia is a country that has invested significant time and effort in the coordination of health sector aid for well over a decade. During this time, much has been achieved in terms of improvement in donor coordination at the national level, both in Zambia and elsewhere. At the district level, however, this study, although limited to Zambia, indicates that so far the Paris Declaration is yet to demonstrate effects. One reason might be the fact that lower levels have not been sufficiently involved in the coordination process; the integration of partners in planning at the national level is not mirrored in similar efforts at the district level. This is probably an effect of both lack of capacity in the districts and partners not being proactive in informing the districts about their upcoming support. In order to improve coordination at the district level partners must improve the predictability of their support. This could be achieved through donors making more long-term pledges of support and through a more active and continuous dialogue between partners and the districts.

Turning global policy into desired local practice is known to be difficult. The 2010 deadline for achieving the targets of the Paris Declaration is fast approaching and it is time for the 130 adherent countries and institutions to accelerate its implementation. This study suggests that more attention needs to be dedicated to decentralized decision making in coordination and planning of donor support.

## Competing interests

The authors declare that they have no competing interests.

## Authors' contributions

JS, conceived the study. JS, BF, KJ, CC and GT all participated in the design of the study. JS carried out data collection and analysis. All authors participated in manuscript writing and have read and approved the final manuscript.
